# T7-lac promoter vectors spontaneous derepression caused by plant-derived growth media may lead to serious expression problems: a systematic evaluation

**DOI:** 10.1186/s12934-022-01740-5

**Published:** 2022-01-28

**Authors:** Daria Krefft, Maciej Prusinowski, Paulina Maciszka, Aleksandra Skokowska, Joanna Zebrowska, Piotr M. Skowron

**Affiliations:** 1 Department of Molecular Biotechnology, Faculty of Chemistry, University of Gdansk, Wita Stwosza 63 street, 80-308 Gdansk, Poland; 2 Department of Physical Education, University of Physical Education and Sport, Gdańsk, Poland

**Keywords:** Microbiological media, Peptone, T7 transcription, T7 promoter, T7-lac promoter, Tabor-Studier, Protein expression, Toxic proteins, *Escherichia coli*

## Abstract

**Background:**

The widespread usage of protein expression systems in *Escherichia coli* (*E. coli*) is a workhorse of molecular biology research that has practical applications in biotechnology industry, including the production of pharmaceutical drugs. Various factors can strongly affect the successful construction and stable maintenance of clones and the resulting biosynthesis levels. These include an appropriate selection of recombinant hosts, expression systems, regulation of promoters, the repression level at an uninduced state, growth temperature, codon usage, codon context, mRNA secondary structure, translation kinetics, the presence/absence of chaperons and others. However, optimization of the growth medium’s composition is often overlooked. We systematically evaluate this factor, which can have a dramatic effect on the expression of recombinant proteins, especially those which are toxic to a recombinant host.

**Results:**

Commonly used animal tissue- and plant-based media were evaluated using a series of clones in pET vector, containing expressed Open Reading Frames (ORFs) with a wide spectrum of toxicity to the recombinant *E. coli*: (i) *gfpuv* (nontoxic); (ii) tp84_28—which codes for thermophilic endolysin (moderately toxic); and (iii) *tthHB27IRM*—which codes for thermophilic restriction endonuclease-methyltransferase (REase-MTase)—RM.TthHB27I (very toxic). The use of plant-derived peptones (soy peptone and malt extract) in a culture medium causes the T7-lac expression system to leak. We show that the presence of raffinose and stachyose (galactoside derivatives) in those peptones causes premature and uncontrolled induction of gene expression, which affects the course of the culture, the stability of clones and biosynthesis levels.

**Conclusions:**

The use of plant-derived peptones in a culture medium when using T7-lac hybrid promoter expression systems, such as Tabor-Studier, can lead to uncontrolled production of a recombinant protein. These conclusions also extend to other, *lac* operator-controlled promoters. In the case of proteins which are toxic to a recombinant host, this can result in mutations or deletions in the expression vector and/or cloned gene, the death of the host or highly decreased expression levels. This phenomenon is caused by the content of certain saccharides in plant peptones, some of which (galactosides) may act as T7-lac promoter inducer by interacting with a Lac repressor. Thus, when attempting to overexpress toxic proteins, it is recommended to either not use plant-derived media or to use them with caution and perform a pilot-scale evaluation of the derepression effect on a case-by-case basis.

**Supplementary Information:**

The online version contains supplementary material available at 10.1186/s12934-022-01740-5.

## Background

Recombinant proteins are indispensable in molecular biology research and biotechnology, industrial and medical applications. A variety of expression systems have been developed, both procaryotic and eucaryotic. The most commonly used recombinant hosts include *E. coli*, *Bacillus subtilis*, *Leishmania tarentolae*, baculovirus and hamster and human cells. *E. coli*-based systems have historically been used most often—they are the most economical, thanks to their rapid growth rate and inexpensive media. Furthermore, *E. coli* is the most ‘worked-out’ organism, meaning that subtle genetic and metabolic aspects which affect the expression of recombinant proteins are known in detail. However, bacterial expression systems have some drawbacks, such as the lack of posttranslational modifications—which are present in eucaryotes—and problems with protein folding. Nevertheless, procaryotic systems are also often used for safety, as the subsequent use of the resulting recombinant products are an important factor. Most restrictions are encountered during the production of substances offered to humans, whether in the form of cosmetics, food, medicine or vaccines. The final product, despite the typical multi-stage purification procedures, may contain traces of substances used in its manufacture that are toxic or may even contain carried over pathogens. One of the most serious types turned out to be those pathogens which can originate from animals, such as viruses and prions. The potential for them to be found in animal products (e.g., the serum or peptones used in culture media) is one reason for the frequent prohibition of their use in bioproduction. This is especially true for bovine products due to the possibility of infection with transmissible spongiform encephalopathy (TSE) [1]. Peptones of animal origin have been successfully replaced with peptones produced from plants, such as soybeans, peas, cottonseeds, rice or wheat. However, they also proved to be a possible source of infection (e.g., mycoplasma) [2]. Therefore, in some cases, chemically defined media are recommended. One of the most commonly used plant peptones comes from soybeans. The content of crude protein in soybeans is about 40%, while in soy flour it is even higher, ranging from 44 to 49% [3]. It is noteworthy that soybeans also contain 30–35% carbohydrates [4, 5]. In the case of soybean meal, this percentage is even higher: reaching about 40% [5]. Due to their physicochemical properties, the plant-derived carbohydrates can be divided into 2 groups. The first group contains structural polysaccharides, which also include dietary fibre components [6]. The latter group consists of nonstructural carbohydrates, i.e., low-molecular-weight sugars, oligosaccharides and storage saccharides [7]. In qualitative terms, nonstructural carbohydrates make up half of the carbohydrates present in soybeans and soybean meal; their concentration in soybean meal oscillates around 20% [8, 9]. The composition of carbohydrates in soy meal is influenced by many factors, such as the technology used in the plant’s processing [9], the variety of soybean used [10], the degree of soybean maturity [11] or even its germination [12]. In addition to sucrose, which is the most abundant in soybeans, research indicates the presence of significant amounts of monosaccharides and oligosaccharides. Low-molecular-weight sugars that are found in both soybeans and soybean meal are sucrose, raffinose, stachyose and verbascose. Monosaccharides such as glucose, galactose, fructose, rhamnose or arabinose are also present in soybeans, but not in soybean meal because they are broken down or removed during processing [13, 14]. As a result, in addition to proteins, peptides and amino acids in soy peptone, there is also a large amount of saccharides, compounds that in some expression systems can act as a gene-inducing factor. This is the case with lactose operon [15], arabinose operon [16], rhamnose operon [17] and maltose operon [18], among others. The best known, historically, is lactose operon. The knowledge learned during research on this operon and modifications to its components have led to a gene expression system that uses a promoter derived from the bacteriophage T7, which is not recognised by the host *E. coli*, but is recognised by specific T7 RNA polymerase. This system was developed in the mid-1980s by 2 independent research teams [19, 20]; since then, it has been widely used and refined [21–23]. Moreover, the T7 polymerase/promoter expression system, originally used in engineered *E. coli* strains, has also been modified and used to overproduce proteins in other bacteria, such as *Streptomyces lividans* [24] or *Bacillus megaterium* [25], in eucaryotes (yeast) [26] and even in mammalian cells [27, 28].

## Materials and methods

### Reagents and media, bacterial strains and plasmids

The media components used in the study were acquired from BTL (Łódź, Poland). Pierce™ Unstained Protein MW Marker, PageRuler™ Plus Prestained Protein Ladder and PageRuler™ Unstained Broad Range Protein Ladder were sourced from Thermo Fisher Scientific Baltics UAB (Vilnus, Lithuania). Marathon DNA polymerase and DNA purification kits were obtained from A&A Biotechnology (Gdynia, Poland). The restriction enzymes (REases) and Antarctic Phosphatase were purchased from New England Biolabs (Ipswich, MA, USA). T4 DNA Ligase was from Epicentre (Madison, WI, USA). The plasmid pGFPuv was purchased from Clontech/Takara Bio Inc. (Kusatsu, Shiga, Japan). *E. coli* DH5α cells [F^−^ φ80*lac*Z∆M15 ∆(*lac*ZYA-*arg*F) U169 *deo*R *rec*A1 *end*A1 *hsd*R17 (r_K_^−^, m_K_^+^) *pho*A *sup*E44 λ^−^ *thi*-1 *gyr*A96 *rel*A1 Mrr-] were used for clone selection (Life Technologies, Carlsbad, CA, USA). For protein expression, *E. coli* BL21(DE3) [F^−^ *omp*T *hsd*SB(r_B_^−^, m_B_^−^) *gal dcm* (DE3)] from Life Technologies (Carlsbad, CA, USA) and the T7 promoter-based pET21d(+) vector from Novagen (Madison, WI, USA) were used. The other reagents and chemicals were sourced from POCh S.A. (Gliwice, Poland), Sigma-Aldrich (St. Louis, MO, USA), Fluka Chemie GmbH (Buchs, Switzerland) or AppliChem Inc. (St. Louis, MO, USA). The oligodeoxyribonucleotide synthesis and DNA sequencing were conducted at Genomed S.A. (Warsaw, Poland).

### Construction of the expression clones

During the study, the vector pET21d(+) was used with cloned genes encoding the proteins GFPuv, RM.TthHB27I and TP84_28 (endolysin). Cloning of the synthetic *tthHB27IRM* gene has been described previously [29]. Cloning and characterization of TP-84 bacteriophage [30] endolysin will be published elsewhere (manuscript in preparation). The gene encoding the protein GFPuv was amplified from the commercially available vector pGFPuv as a result of PCR carried out in the presence of a primer pair—forward: 5′-GGGGTCATGAGTAAAGGAGAAGAACTTTTCACTGGA-3′ and reverse: 5′-CCAGACAAGTTGGTAATGGTAGCGAC-3′. The reactions were performed in 100-µl volumes in a 2720 thermal cycler (Perkin Elmer Applied Biosystems) and contained 50 ng of template DNA, 1 ×  Marathon PCR reaction buffer, 0.5 µM of each primer, 0.4 mM of dNTPs and 1.5 units of Marathon DNA polymerase. The cycling profile consisted of the following stages: 97 °C for 2 min 30 s, then 40 cycles of 94 °C for 30 s, 55 °C for 30 s and 72 °C for 30 s, and a final elongation step of 72 °C for 1 min 30 s. The resulting PCR product was cleaved with EcoRI and BspHI REases and purified using a Clean-Up AX kit. The vector pET21d(+) was cleaved with NcoI and EcoRI REases, dephosphorylated and gel purified using a Gel-Out AX kit. The prepared vector and insert were ligated; the reaction mixture was then phenol–chloroform extracted and ethanol precipitated. The purified DNA was used to transform electrocompetent *E. coli* DH5α cells. The bacteria were grown on Petri dishes with LA medium [31] supplemented with ampicillin (100 μg/ml) at 30 °C. The resulting test clones were analysed with ScaI REase and then sequenced. The DNA from positive clones was used to transform the expression *E. coli* BL21(DE3) strain cells.

### Culturing of the clones in the E. coli BL21(DE3) in tested growth media and evaluation of selected saccharides

As a result of electroporation, *E. coli* BL21(DE3) cells carrying all 3 plasmids were obtained. *E. coli* BL21(DE3) [pET21d(+)-*gfpuv*], BL21(DE3) [pET21d(+)-*tthHB27IRM*] and BL21(DE3) [pET21d(+)-*TP84_28*] liquid cultures were performed analogously. A M9 minimal medium [32] and 9 variants of LB broth [31] were used to carry out the cultures, where the nitrogen source varied. The control culture was carried out in a medium containing only 0.5% yeast extract and 1% NaCl. In subsequent cultures, third media components were added in amounts of 1%: soy peptone, malt extract, casein peptone, gelatin peptone, tryptone peptone, tryptose peptone, proteose peptone and peptobak. The M9 minimal medium was prepared according to the Sambrook method [32]. For the solid media, agar was added to 1.5%. All media were supplemented with 100 µg/ml of ampicillin. The solid cultures were carried out at 30 °C for 24 h. The liquid cultures were conducted in 100 ml of each medium at 37 °C with vigorous aeration for 24 h. When the cultures reached the optical density OD_600_  = 0.6–0.8, samples were taken for spectrophotometric measurements and SDS-PAGE analysis, at an interval of 1 h for 6 consecutive hours and after an overnight incubation. Measurements were started at OD_600_  = 0.6–0.8, as this is the value at which it is recommended to induce gene expression in the Tabor-Studier system [33]. For the evaluation of the selected saccharides, which are naturally present in plant-based media, gene expression in *E. coli* BL21(DE3) [pET21d(+)-*gfpuv*] was used as a test system. Cultures were grown at 37 °C in 100 ml of LB medium based on the least autoinducing component—tryptone peptone—supplemented with 100 µg/ml of ampicillin. When the OD_600_ reached 0.6–0.8, a sterile solution of one of the saccharides being tested was added to the culture to a final concentration of 1 mM and the cultures were further grown for 7 h. Samples were taken every hour for spectrophotometric measurements and analysis by glycine-based SDS-PAGE [34] on 7.5–10% polyacrylamide gels, further stained using Coomassie Brilliant Blue R-250.

## Results and discussion

### The effect of selected peptones on leakage in the T7-lac expression system

**Fig. 1:**
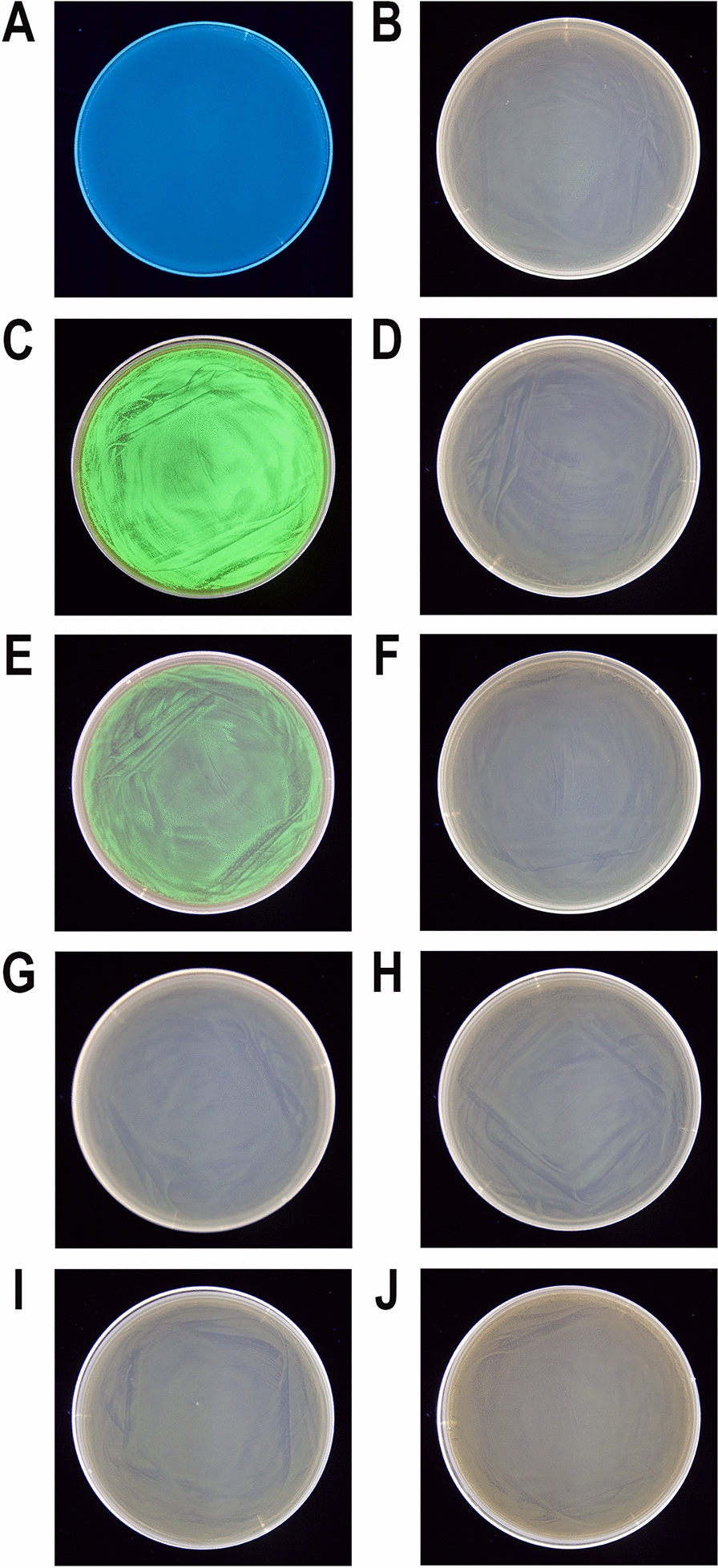
Fluorescence of *E. coli* BL21(DE3) [pET21d(+)-*gfpuv*] bacterial colonies on solid media with various compositions. *E. coli* BL21(DE3) bacteria were transformed with the plasmid pET21d(+)-*gfpuv* and plated on Petri dishes with solid M9 minimal medium and 9 variants of LA solid medium containing different nitrogen sources. Cultures were grown overnight at 30 °C. **A** solid M9 minimal medium—this medium did not contain UV/blue-adsorbing organic content, so it was translucent to the transilluminator’s wavelength spectrum; **B** medium consisting of only yeast extract, NaCl and agar; **C** as in **B** with the addition of soya peptone; **D** tryptone peptone; **E** malt extract; **F** tryptose peptone; **G** gelatin peptone; **H** casein peptone; **I** proteose peptone; and **J** peptobak

**Fig. 2:**
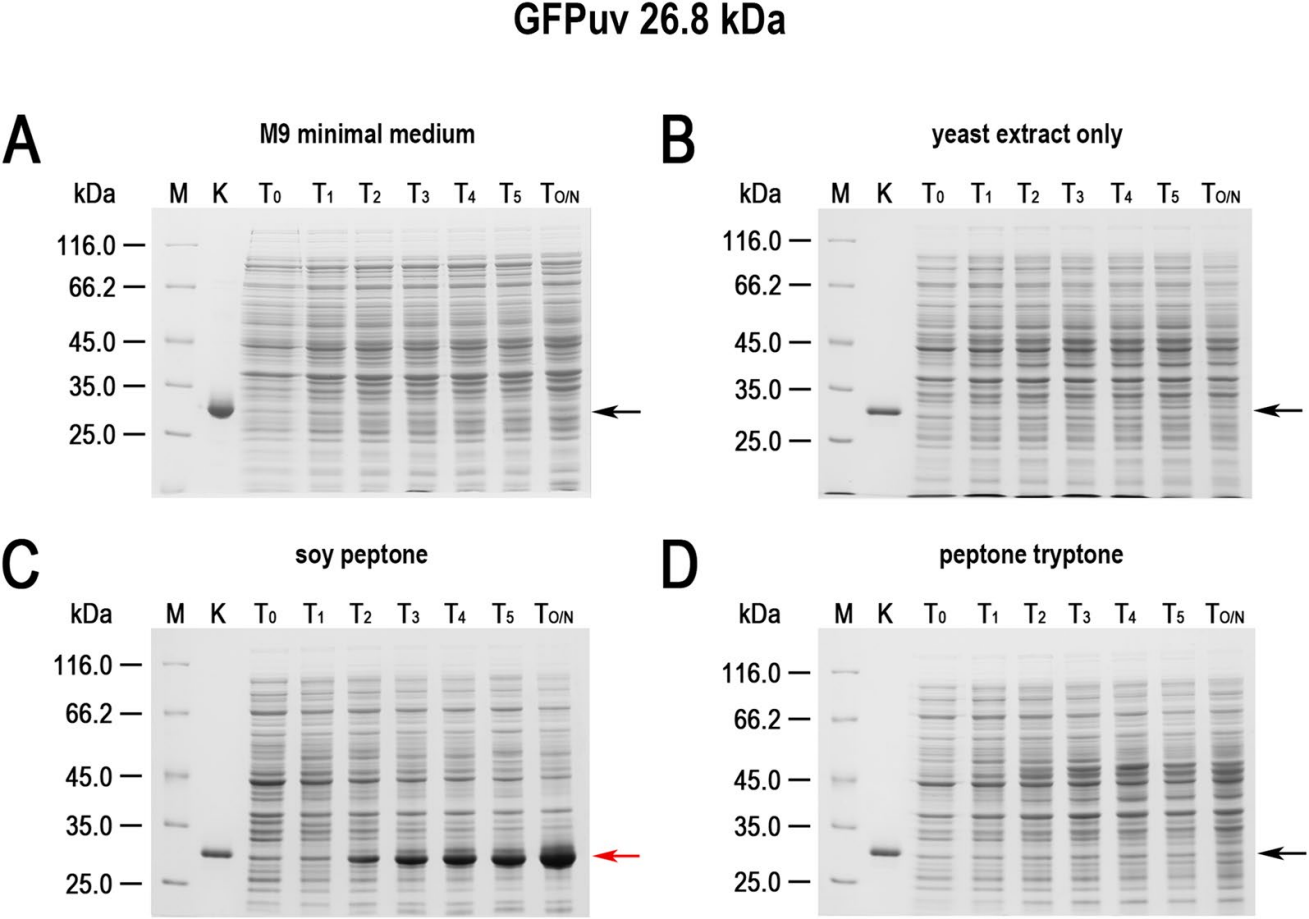
Expression leakage of GFPuv in *E. coli* BL21(DE3) [pET21d(+)-*gfpuv*] cells on media of various compositions. The cultures were grown at 37 °C in **A** M9 minimal medium and variants of LB medium. The tested LB media contained yeast extract and NaCl, supplemented with the tested peptones; **B** control culture, grown in a medium without a peptone; **C** medium with soya peptone added; and **D** tryptone peptone. Samples were taken at 1-h intervals, starting when the cultures reached OD_600_  = 0.6–0.8, and were then subjected to spectrophotometric and SDS-PAGE analysis. Bacterial cells were lysed and analysed using a 10% gel. (Lane M) Pierce™ Unstained Protein MW Marker (Thermo Scientific); (Lane K) purified recombinant GFPuv protein; (Lane T_0_) *E. coli* BL21(DE3) [pET21d(+)-*gfpuv*] cells at OD_600_  = 0.6–0.8; (Lane T_1_) cells 1 h after the culture reached OD_600_  = 0.6–0.8; (Lane T_2_) 2 h; (Lane T_3_) 3 h; (Lane T_4_) 4 h; (Lane T_5_) 5 h; and (Lane T_O/N_) cells after overnight cultivation. The arrows indicate the position (26.8 kDa) at which GFPuv migrates in the gel. The red arrow indicates GFPuv in the samples containing the expressed *gfpuv* gene

**Fig. 3:**
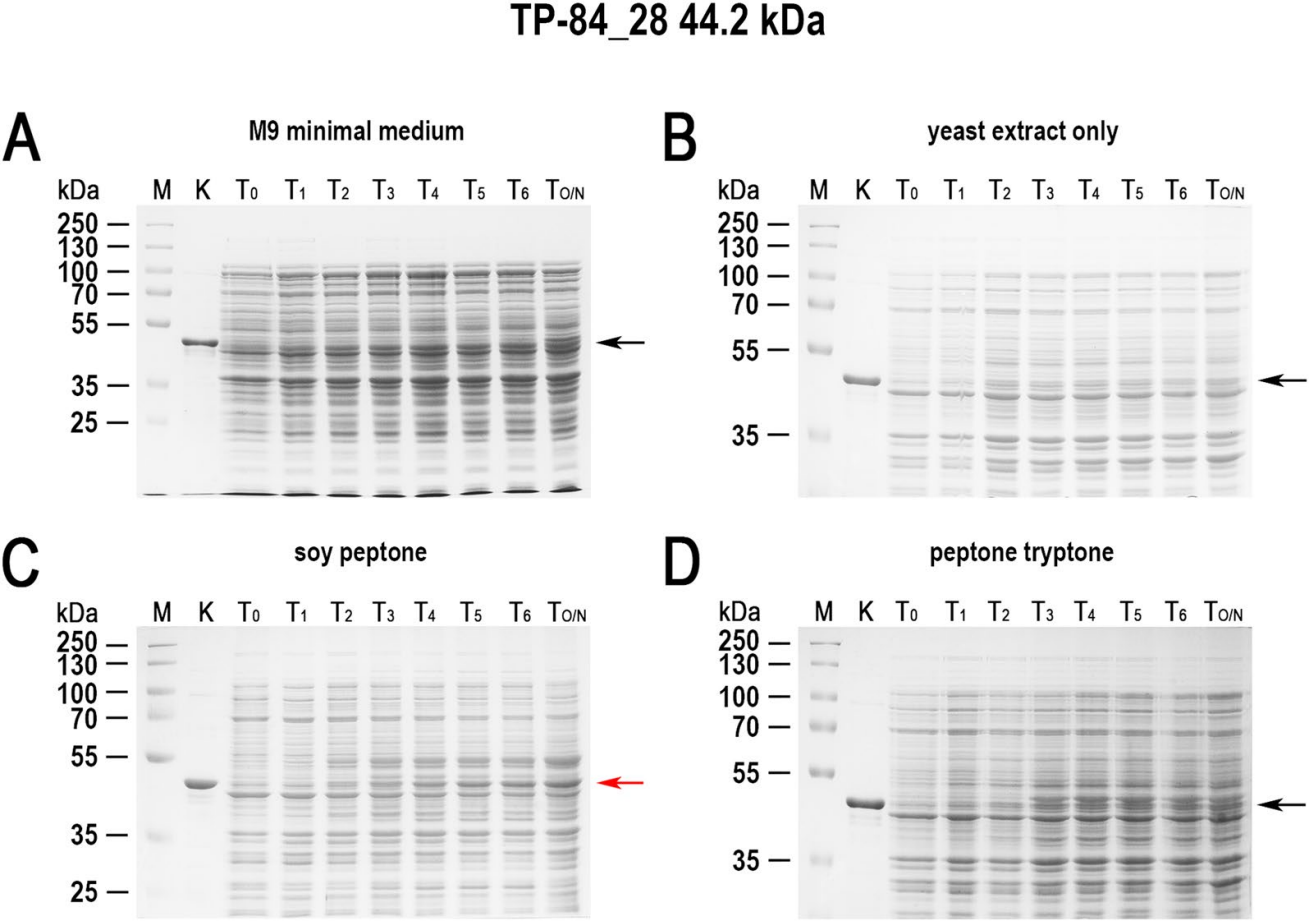
Expression leakage of TP-84 endolysin in *E. coli* BL21(DE3) [pET21d(+)-*tp84_28*] cells on the media with various compositions. The cultures were carried out as for *E. coli* BL21(DE3) [pET21d(+)-*gfpuv*]. The bacterial cells were lysed and analysed on 10% SDS-PAGE. (Lane M) PageRuler™ Plus Prestained Protein Ladder (Thermo Scientific); (Lane K) purified recombinant TP84_28 protein; (Lane T_0_) *E. coli* BL21(DE3) [pET21d(+)-*tp84_28*] cells at OD_600_  = 0.6–0.8; (Lane T_1_) cells 1 h after the culture reached OD_600_  = 0.6–0.8; (Lane T_2_) 2 h; (Lane T_3_) 3 h; (Lane T_4_) 4 h; (Lane T_5_) 5 h; (land T_6_) 6 h; and (Lane T_O/N_) cells after overnight cultivation. The arrows indicate the position (44.2 kDa) at which endolysin migrates in the gel. The red arrow indicates TP84_28 in the samples containing the expressed *tp84_28* gene

**Fig. 4:**
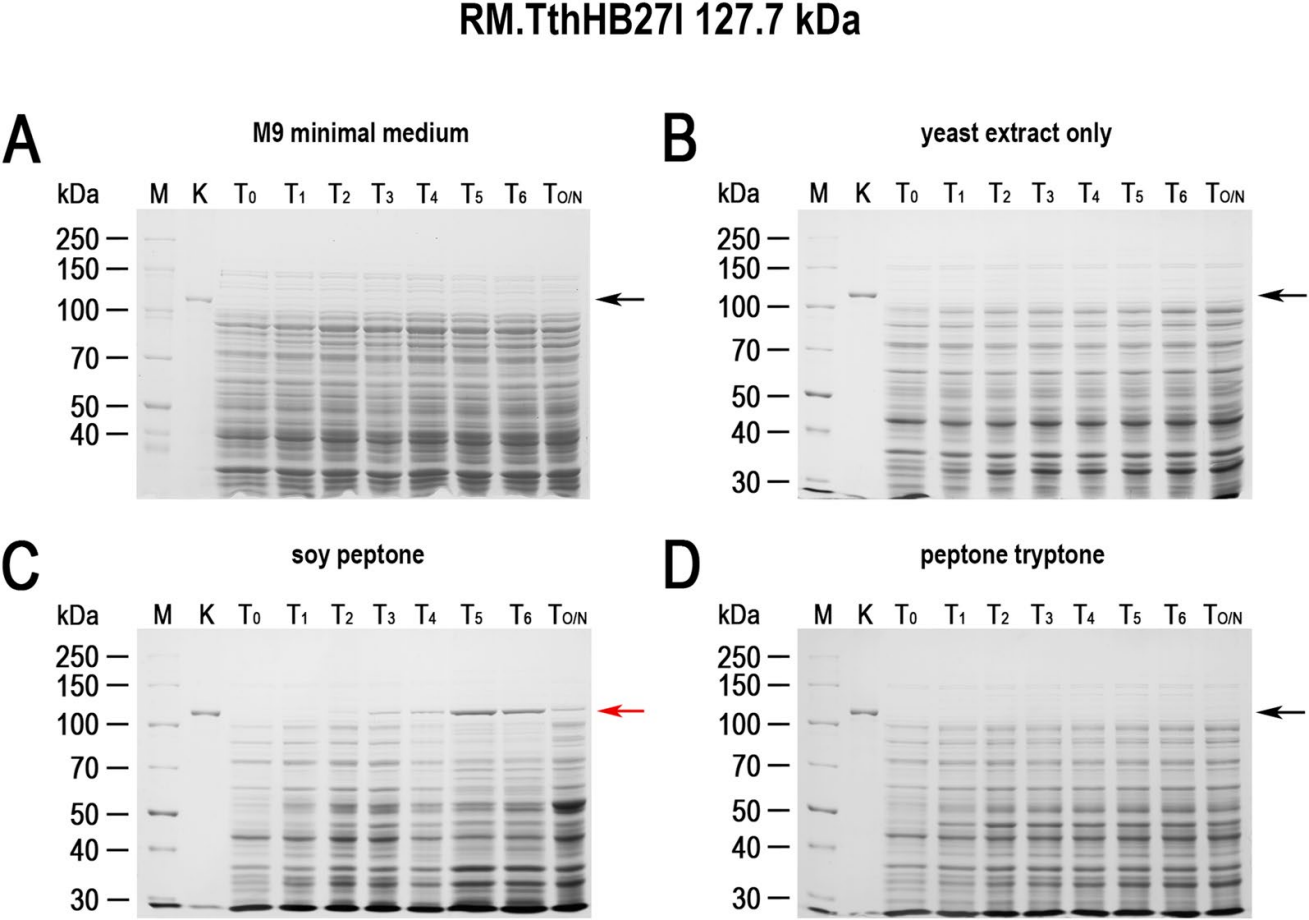
Expression leakage of RM.TthHB27I in *E. coli*. The cultures were carried out as for *E. coli* BL21(DE3) [pET21d(+)-*gfpuv*]. The bacterial cells were lysed and analysed on 7.5% SDS-PAGE. (Lane M) PageRuler™ Unstained Broad Range Protein Ladder (Thermo Scientific); (Lane K) purified recombinant RM.TthHB27I protein; (Lane T_0_) *E. coli* BL21(DE3) [pET21d(+)-*tthHB27IRM*] cells at OD_600_ = 0.6–0.8; (Lane T_1_) cells 1 h after the culture reached OD_600_ = 0.6–0.8; (Lane T_2_) 2 h; (Lane T_3_) 3 h; (Lane T_4_) 4 h; (Lane T_5_) 5 h; (land T_6_) 6 h; and (Lane T_O/N_) cells after overnight cultivation. The arrows indicate the position (127.7 kDa) at which RM.TthHB27I migrates in the gel. The red arrow indicates RM.TthHB27I in the samples containing the expressed *tthHB27IRM* gene

**Fig. 5:**
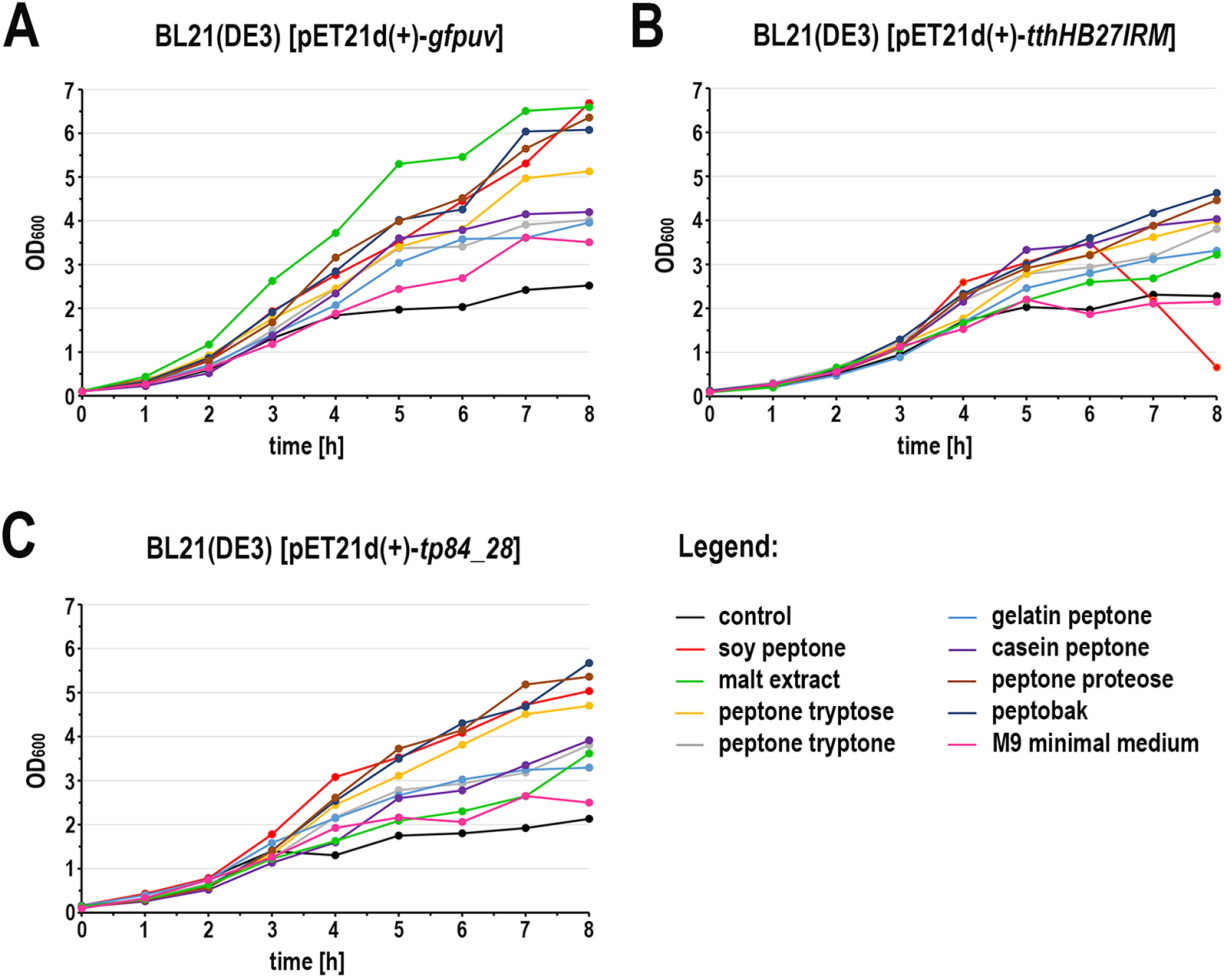
Media composition effect on the cultures’ growth kinetics. The growth kinetics of the *E. coli* BL21(DE3) [pET21d(+)-*gfpuv*], *E. coli* BL21(DE3) [pET21d(+)-*tthHB27IRM*] and *E. coli* BL21(DE3) [pET21d(+)-*tp84_28*] bacterial cultures in M9 minimal medium and LB media supplemented with different peptones was measured as OD_600_. The cultures were carried out at 37 °C. No gene expression-inducing factors were added during culturing

While conducting various cultures of recombinant *E. coli*, aimed at expressing cloned genes in the Tabor-Studier system (pET-series), we observed that despite identical growth conditions, the cultures sometimes behaved differently. This concerned both the cultures grown under non-inducing conditions as well as upon induction to the overproduced recombinant proteins. After analysing the series of control experiments (not shown), the only factor differentiating the erratically behaving *E. coli* cultures, carrying the same genetic constructs, was the use of soy peptone or tryptone peptone during the preparation of culture media. To further investigate this phenomenon, a number of *E. coli* BL21(DE3) cultures carrying one of genes *gfpuv, tthHB27IRM* or *tp84_28* were performed. These test genes were selected to cover a wide range of toxicity to the recombinant *E. coli*: (i) *gfpuv* (nontoxic); (ii) tp84_28—which codes for thermophilic endolysin (moderately toxic); and (iii) *tthHB27IRM*—which codes for thermophilic RM.TthHB27I (very toxic). The protein GFPuv, which is very well tolerated by *E. coli,* has an additional advantage in our expression evaluation, in that it can be assayed in whole cells not only by SDS-PAGE electrophoretic analysis, but also by simply exposing the bacteria to UV light; thus, it is well suited for this study. The second protein selected for analysis was the TP-84 bacteriophage endolysin, which executes a moderate toxic effect on host cells. It is not problematic to maintain its pET vector-based expression clone in an uninduced state, while upon induction the recombinant *E. coli* cells become fragile and prone to spontaneous lysis, apparently due to a penetration of small amounts of the protein through the cytoplasmatic membrane and a degradation of the peptidoglycan layer. The third protein selected for the study was the recombinant RM.TthHB27I, originating from *Thermus thermophilus* HB27I. Due to its unbalanced REase versus MTase activities in the recombinant *E. coli* host cells, it is very toxic and frequently causes recombinant *E. coli* cells to lyse or mutants to accumulate during its induced expression. Sometimes, those problems occurred even in the uninduced state [29, 35]. Thus, to obtain an adequate biosynthesis level of the protein RM.TthHB27I with biological activity, it is essential to maintain a mutant-free *E. coli* population that does not die prematurely and that carries the pET vector-based expression construct by exercising strict control of the T7-lac promoter. For overproduction of the test proteins, *E. coli* BL21(DE3) cells were transformed with the plasmids pET21d(+)-*gfpuv*, pET21d(+)-*tthHB27IRM* and pET21d(+)-*tp84_28*, then plated on solid M9 minimal medium and the 9 variants of LA medium described below before being incubated overnight at 30 °C. At higher incubation temperatures, no or very few transformants carry the *tthHB27IRM* gene [29]. In the case of the endolysin clone, incubations at both 30 °C and 37 °C resulted in appropriate colonies, but 30 °C was used as a precaution. For GFPuv, no toxic effect was observed, but overproduction of the protein at lower temperatures is advisable, as it is better suited for proper GFP protein folding and solubility [36]. The media variants contained different types of peptones, or no peptones in the case of the control culture. No IPTG or other gene expression inducer acting on the T7-lac promoter was added. After overnight incubation, bacterial colonies were observed on plates with all media (for all expression plasmids). The plates with bacteria carrying the plasmid pET21d(+)-*gfpuv* were exposed to UV light using a transilluminator (Fig. 1). As clearly seen in Fig. 1C, the bacteria that grew on the LA medium made with soy peptone showed a strong green fluorescence, which indicates the presence of GFPuv protein overexpression. Green fluorescence was also observed in the case of the malt extract substrate (Fig. 1E); however, its level was significantly lower. Fluorescence was not observed for bacteria that grew on the other media tested (Fig. 1A, B, D, F–J), which indicates no or very low T7-lac promoter leakage. Nowadays, when expressing cloned genes, constitutive promoters are rarely used. To the contrary, typically, expression systems with a strict process control are used. However, due to the imperfections associated with each expression system, a number of methods have been developed to increase their tightness. In the case of protein overproduction in the Tabor-Studier system, these include (i) co-expression of the gene encoding the protein of interest with an additional copy of the gene encoding the LacI repressor; (ii) the gene coding for T7 lysozyme, which is an inhibitor of T7 RNA polymerase; and (iii) introduction of the *lac*O operator behind the T7 promoter sequence, forming a fusion T7-lac promoter [37]. Also, decreasing the temperature to even below 20 °C for certain engineered *E. coli* strains helps to control recombinant protein deleterious activity. Even a low level of background expression can have deplorable effects from the overproduction of toxic proteins: a slower growth rate of the culture, less protein biosynthesis, mutants accumulation, plasmid loss, low culture cell density or cell death [38, 39]. To illustrate this effect, *E. coli* BL21(DE3) cultures carrying the 3 test plasmids in liquid media of different compositions (analogously to the cultures on solid media) were performed. The SDS-PAGE analysis of the cellular proteins profile at individual stages of the cultures showed that the increase in GFPuv biosynthesis (Fig. 2; Additional file 1) can be observed in uninduced cultures on a medium containing either soy peptone or malt extract. The amount of GFPuv protein produced in both cases was very different—using soy peptone resulted in a massive expression of GFPuv, visible as a dominant band (26.8 kDa, marked as red arrows) on SDS-PAGE (Fig. 2C; Additional file 1A). However, in the case of other peptones, no proteins corresponding to the control protein size (purified GFPuv preparation) were observed (Fig. 2A, B, D; Additional file 1B–F). This result coincides very well with the results of the culture carried out on solid media, though more information was obtained concerning the effect of uncontrolled protein overproduction. A somewhat similar effect to GFPuv was observed during the SDS-PAGE analysis of the profile of cellular proteins at individual stages of culturing, producing an endolysin (44.2 kDa, red arrows) clone, although the level of protein biosynthesis was much lower (Fig. 3; Additional file 2). The situation was different in the case of cultures carrying the plasmid pET21d(+)-*tthHB27IRM* (Fig. 4; Additional file 3). The appearance of a protein was observed of a size corresponding to the control protein (purified RM.TthHB27I preparation, 127.7 kDa) in the culture grown in the presence of soy peptone (Fig. 4C). The analysis of the growth curves of individual cultures (Fig. 5; Additional file 4) led to several observations. The control cultures in all cases exhibited weaker growth, which had been expected due to the limited source of nutrients (yeast extract only). The cultures grown in the media containing various peptones of animal origin showed similar growth kinetics. However, the most striking was the comparison of the course of all cultures carried out in the media containing plant-derived peptones. For *E. coli* BL21(DE3) [pET21d(+)-*tthHB27IRM*] cultures (Fig. 5B), a drastic decrease in cell density and spontaneous cells lysis (between 6 and 8 h of cultivation) was observed in the media containing soy peptone, typical for T7-lac promoter-induced cultures overproducing proteins that are toxic to *E. coli* host cells. This result coincided with a large increase in RM.TthHB27I protein biosynthesis in the cells, as seen on the SDS-PAGE gel (Fig. 4C, samples for points T_5_ and T_6_). The optical density of the *E. coli* BL21(DE3) [pET21d(+)-*tthHB27IRM*] and *E. coli* BL21(DE3) [pET21d(+)-*tp84_28*] cultures—grown in the presence of malt extract—was the lowest among those grown in a medium with complete composition. To the contrary, *E. coli* BL21(DE3) [pET21d(+)-*gfpuv*] producing nontoxic GFPuv showed the fastest growth on the malt extract, apparently due to the presence of large amounts of saccharides, serving as a rich energy source. In the case of the *E. coli* BL21(DE3) [pET21d(+)-*gfpuv*] or *E. coli* BL21(DE3) [pET21d(+)-*tp84_28*] cultures (Fig. 5A, C), no decrease in density was observed in the cultures grown in soy peptone media, even though it also apparently contained some T7-lac promoter-inducing components, as shown in Fig. 1. Moreover, these cultures displayed very high optical density values on soy peptone, which indicates favourable nutritional conditions for the tested bacteria. However, when the malt extract was present in the culture medium, the bacteria carrying the gene that encodes endolysin behaved similarly to the bacteria carrying the gene that encodes RM.TthHB27I: the growth was relatively slow, but the cells evidently managed to cope with small amounts of those toxic proteins. On the other hand, the bacteria carrying the gene that codes for the protein GFPuv behaved exactly the opposite in this medium (Fig. 5A). The presence of malt extract caused a very rapid increase in biomass in the *E. coli* BL21(DE3) [pET21d(+)-*gfpuv*] culture, and the optical density obtained after overnight incubation was 2–4 times higher than in other cultures. This result differs from those presented by other researchers, showing that the overproduction of even a nontoxic protein in such massive amounts usually places a heavy strain on cell metabolism, which should rather adversely affect the development of the culture [40, 41]. Table 1 shows a quantitative presentation of these findings, based on a comparison of model, non-toxic protein GFPuv expression levels in various media, grown for up to 5 h, as recommended for the pET system. GFPuv was selected in order to avoid an interference of the toxic effect of TP-84 endolysin and RM.TthHB27I on expression. Strikingly, soy peptone is nearly as effective as IPTG in inducing GFPuv biosynthesis. While it suggests that this medium should be avoided in the case of cultivating recombinant clones expressing toxic proteins, it can be used a cost-effective inducer in place of IPTG. It also points to the conclusion that the metabolic stress highly depends on a given protein being overexpressed, which may be useful in optimising biotechnological processes. Other factors taken into account when planning a microbiological culture are the nutritional requirements of a given microorganism and the cost of the components of microbiological media. While *E. coli* is not a very demanding microorganism and grows well on basic media, such as M9 minimal medium or LB, other microorganisms need to be provided with specialised media components, including appropriately selected peptones. An example comparison of the costs and compositions of the individual peptones used in this work is shown in Additional file 5.

**Table 1:** Comparison of GFPuv expression levels in various media (% of IPTG control)

Percent of GFPuv expression						
Time (h)^a^	T_0_	T_1_	T_2_	T_3_	T_4_	T_5_
IPTG^b^ control (control)	15.8	39.2	65.3	83	88.7	100
Soy peptone	12.3	15.1	27.2	54	63.9	94.4
M9 minimal medium	ND	ND	ND	ND	ND	ND
Yeast extract only	ND	ND	ND	ND	ND	ND
Malt extract	ND	ND	ND	ND	ND	ND
Tryptone peptone	ND	ND	ND	ND	ND	ND
Tryptose peptone	ND	ND	ND	ND	ND	ND
Gelatin peptone	ND	ND	ND	ND	ND	ND
Casein peptone	ND	ND	ND	ND	ND	ND
Proteose peptone	ND	ND	ND	ND	ND	ND
Peptobak	ND	ND	ND	ND	ND	ND

*ND* not detectable using densitometry of stained SDS-PAGE gels

^a^Starting from OD_600_  = 0.6–0.8

^b^IPTG-induced control in tryptone peptone-based medium

### The effect of saccharides contained in soy peptones in the Tabor-Studier system

**Fig. 6:**
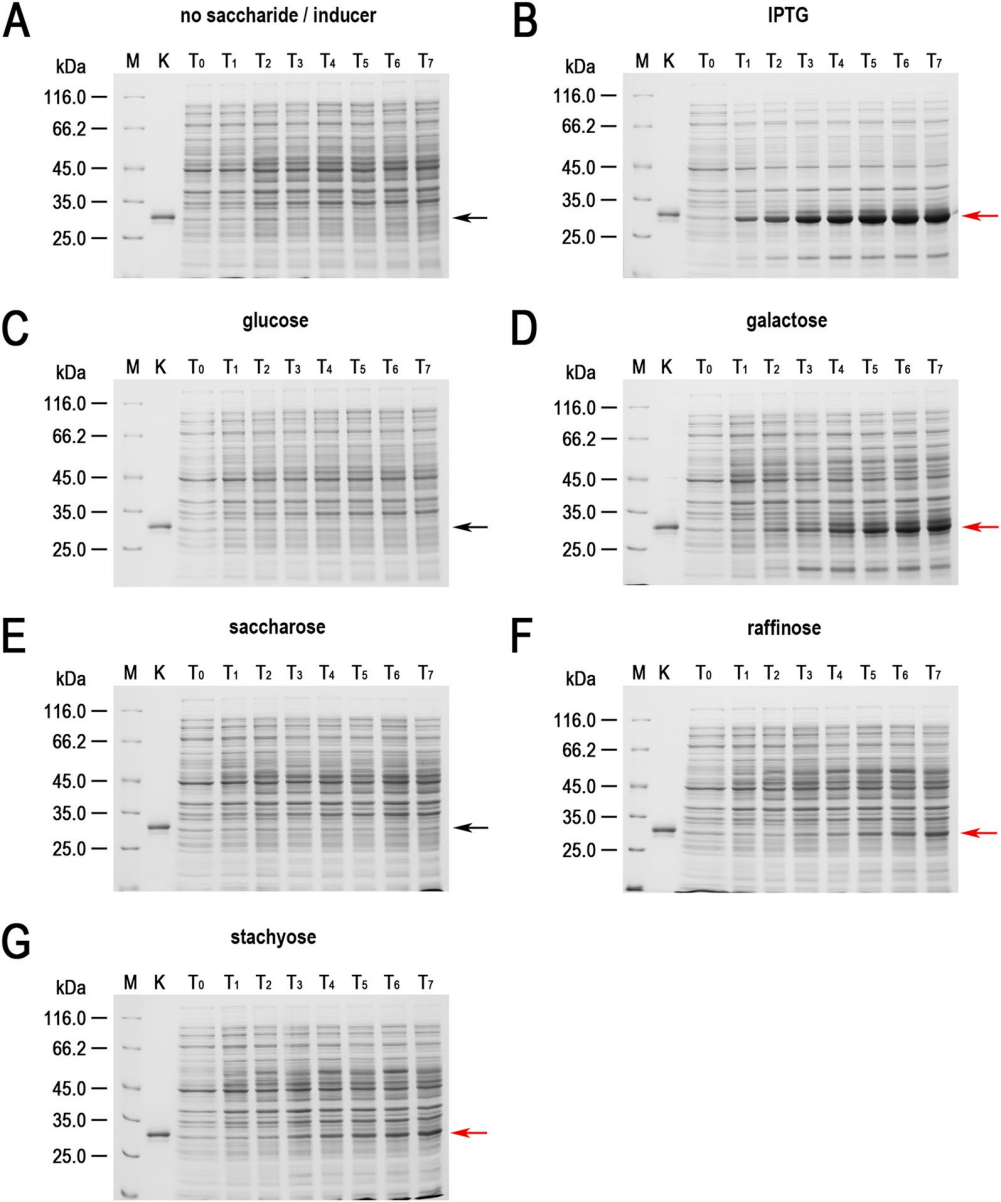
The effect of adding selected saccharides to the culture medium on GFPuv-coding gene expression in the Tabor-Studier system. The cultures were grown at 37 °C in LB medium containing tryptone (a non-inducing animal-based component). When the culture reached OD_600_  = 0.6–0.8, one of the tested saccharides was added to a final concentration of 1 mM: **A** negative control culture, no saccharide added; **B** positive control culture, IPTG added; **C** glucose; **D** galactose; **E** saccharose; **F** raffinose; and **G** stachyose. The culture samples were taken at 1-h intervals starting from the moment the culture reached OD_600_  = 0.6–0.8 and were subjected to spectrophotometric and 10% SDS-PAGE analysis. (Lane M) Pierce™ Unstained Protein MW Marker (Thermo Scientific); (Lane K) purified recombinant GFPuv protein; (Lane T_0_) *E. coli* BL21(DE3) [pET21d(+)-*gfpuv*] cells at OD_600_ = 0.6–0.8; (Lane T_1_) cells 1 h after the culture reached OD_600_ = 0.6–0.8; (Lane T_2_) 2 h; (Lane T_3_) 3 h; (Lane T_4_) 4 h; (Lane T_5_) 5 h; (Lane T_6_) 6 h; and (Lane T_7_) 7 h. The arrows indicate the position (26.8 kDa) at which GFPuv migrates in the gel. The red arrows indicate GFPuv in the samples containing the expressed *gfpuv* gene

**Fig. 7:**
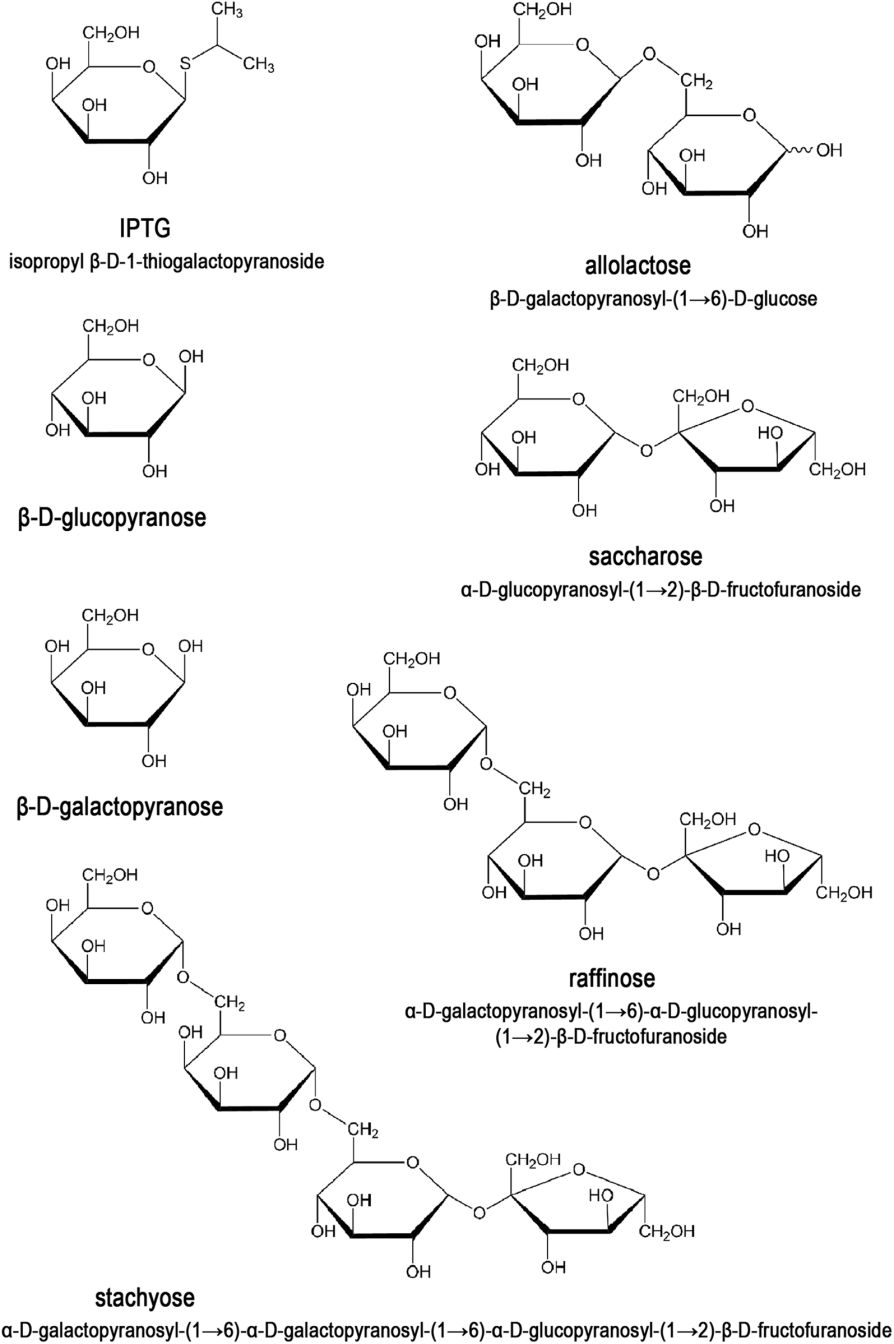
The structures of the gene expression-inducing compounds in the Tabor-Studier system used in this work. The names and structures presented in the Haworth projection of the compounds that induce the expression of *lac* operon genes (IPTG and allolactose) and the saccharides present in significant amounts in soybean meal (glucose, galactose, sucrose, raffinose and stachyose)

Six of the tested peptones (gelatin peptone, casein peptone, tryptone peptone, tryptose peptone, proteose peptone and peptobak) are obtained through the enzymatic digestion of proteins of animal origin. They mainly contain amino acids, peptides and proteins as well as small amounts of the non-inducing T7-lac promoter saccharide, glycogen. On the other hand, soy peptone, obtained from soybean meal, is of plant origin. The origin of this peptone has a critical impact on its composition, as plants typically contain large amounts of various carbohydrates as a storage material in addition to amino acids, peptides and proteins [42, 43]. The main soluble carbohydrates found in soybean meal (and soy peptone) are sucrose, raffinose, stachyose and verbascose. Because verbascose accounts for less than 0.5% of the dry weight of soybean meal [13], it was omitted in further studies. In order to determine which of the above-mentioned carbohydrates may cause leakage in the T7-lac promoter expression system, as well as for the clarity of those experiments, the nontoxic protein producer *E. coli* BL21(DE3) [pET21d(+)-*gfpuv*] was grown in LB medium with tryptone peptone as a nitrogen source and supplemented with the sugar under study at a final concentration of 1 mM when the culture reached OD_600_  = 0.6–0.8. The final concentration of the added sugar solution and the optical density of the culture at which it was added followed the standard conditions for inducing gene expression using IPTG (a synthetic gene expression inducer for the T7-lac expression system). Research on the induction of gene expression began in the 1950s [44]. Since then, it has been shown that β-D-galactosides must be used for promoter induction, controlled by a *lac* operator, and a number of substances have been synthesized that not only do not undergo hydrolysis and metabolism, but are more effective, such as IPTG [45]. Two control cultures were carried out: a negative control, where no additional substance was added, and a positive control, to which IPTG was added. Solutions of 5 carbohydrates—sucrose, raffinose, stachyose, glucose and galactose—were added to other cultures. The last 2 sugars are the monomers that make up allolactose, a naturally occurring compound that induces gene expression from a natural *lac* promoter and from the engineered T7-lac expression system. They are not present in soybean meal and thus are also lacking in soy peptone, but it was important to confirm or exclude their gene expression-inducing effect. Cultures were grown at 37 °C for 7 h from the moment they reached OD_600_  = 0.6–0.8, and samples were taken every hour for spectrophotometric and SDS-PAGE analysis. The profile of cellular proteins at the individual stages of each culture is shown in Fig. 6. In the control (negative) culture, no increase in the protein corresponding to GFPuv over culturing time was observed (Fig. 6A). The cultures with added glucose and sucrose yielded the same results (Fig. 6C, E). An increase of GFPuv protein biosynthesis in bacterial cells was observed for other cultures (Fig. 6B, D, F, G). As expected, the largest increase in protein was found in the control (positive) culture, where IPTG was added (Fig. 6B). The biosynthesis level of GFPuv protein in the culture with the addition of galactose (Fig. 6D) was also significant—7 h after the addition of galactose the amount was comparable to that obtained 2 h after induction with IPTG (Fig. 6B). In the case of raffinose and stachyose, the observed increase in GFPuv protein in the cells was lower than for galactose (Fig. 6F, G). These results indicate that raffinose and stachyose, present in soy peptone, are responsible for a recombinant protein biosynthesis leakage in T7-lac based expression systems, such as the Tabor-Studier system. The structural formulas of these saccharides are shown in Fig. 7. Both raffinose and stachyose are β-D-galactosides, which corroborates the previous finding that various β-D-galactosides can be potential inducers of *lac* operator-controlled promoters. While we have evaluated a limited number of plant-derived media, it is expected that similar effects of leakage in *lac* operator-controlled expression systems also concern other plant-derived peptones (e.g., malt, wheat, rice, peas, cotton or potatoes), as carbohydrates are not completely removed during their production from plant tissues. However, due to differences in the content of individual plant components depending on their species, origin, degree of maturity and processing technology, the effect will certainly vary. Thus, small-scale, individual testing prior to scaled-up production is recommended. In the cases of very toxic recombinant protein production in the Tabor-Studier system or other lac operator-controlled systems, it may become necessary to exclude plant-derived peptones and/or supplement them with other control circuits, such as the co-expression of T7 lysozyme.

## Conclusions

1. When planning a recombinant gene expression it is important to estimate the potential toxicity of the resulting recombinant protein on the recombinant host, due to the inherent leakage of the promoters being used and, accordingly, to select an expression system and media components.
2. Even the most commonly used T7-lac expression systems, considered to be tightly controlled, are prone to massive leakage on some growth media, due to the specific recognition by T7 RNA polymerases.
3. The animal-origin media tested herein (gelatin peptone, casein peptone, tryptone peptone, tryptose peptone, proteose peptone and peptobak) did not cause leakage, while the plant-origin media (soy peptone and malt extract) resulted in massive, uncontrolled recombinant gene expression in an uninduced state which, in the case of the toxic protein RM.TthHB27I, led to culture cell lysis.
4. Various saccharides that are typically present in plant tissues were examined for their undesirable induction of T7-lac promoter; it was found that β-D-galactosides—galactose, raffinose and stachyose—are inducers, while glucose and saccharose are not.

## Supplementary Information

**Additional file 1: **Expression leakage of GFPuv in *E. coli* BL21(DE3) [pET21d(+)-*gfpuv*] cells grown in media containing selected peptones. The cultivation, sample preparation and analysis were identical to that described in Fig. 2, except that a different peptone was supplemented in the composition of the medium instead of the most commonly used soya peptone and tryptone peptone: **A** wheat extract; **B** tryptose peptone; **C** gelatin peptone; **D** casein peptone; **E** proteose peptone; and **F** peptobak.

**Additional file 2: **Expression leakage of TP-84 endolysin in *E. coli* BL21(DE3) [pET21d(+)-*tp-84_28*] cells grown in media containing selected peptones. The cultivation, sample preparation and analysis were identical to those described in Fig. 3, except that a different peptone was supplemented in the composition of the medium instead of the most commonly used soya peptone and tryptone peptone: **A** wheat extract; **B** tryptose peptone; **C** gelatin peptone; **D** casein peptone; **E** proteose peptone; **F** peptobak.

**Additional file 3: **Expression leakage of RM.TthHB27I in *E. coli* BL21(DE3) [pET21d(+)-*tthHB27IRM*] cells grown in media containing selected peptones. The cultivation, sample preparation and analysis were identical to that described in Fig. 4, except that a different peptone was supplemented in the composition of the medium instead of the most commonly used soya peptone and tryptone peptone: **A** wheat extract; **B** tryptose peptone; **C** gelatin peptone; **D** casein peptone; **E** proteose peptone; **F** peptobak.

**Additional file 4: **Bacterial culture optical density measurements data. Optical density measurements were performed at a wavelength of 600 nm using sterile medium as a background.

**Additional file 5: **Table summarising the basic data on the tested nutrients and price comparison. The price comparison is shown as of the highest-priced component. The prices quoted by the producers of the ingredients were used in this research may vary.

## Data Availability

All data generated or analysed during this study are included in this published article. The datasets used and/or analysed during the current study are available from the corresponding author on reasonable request.
