# Supplementary figures and images for: T7-lac promoter vectors spontaneous derepression caused by plant-derived growth media may lead to serious expression problems: a systematic evaluation
Source: Microb Cell Fact. 2022 Jan 28;21:13. doi: 10.1186/s12934-022-01740-5 (PMC8796431; doi:10.1186/s12934-022-01740-5)

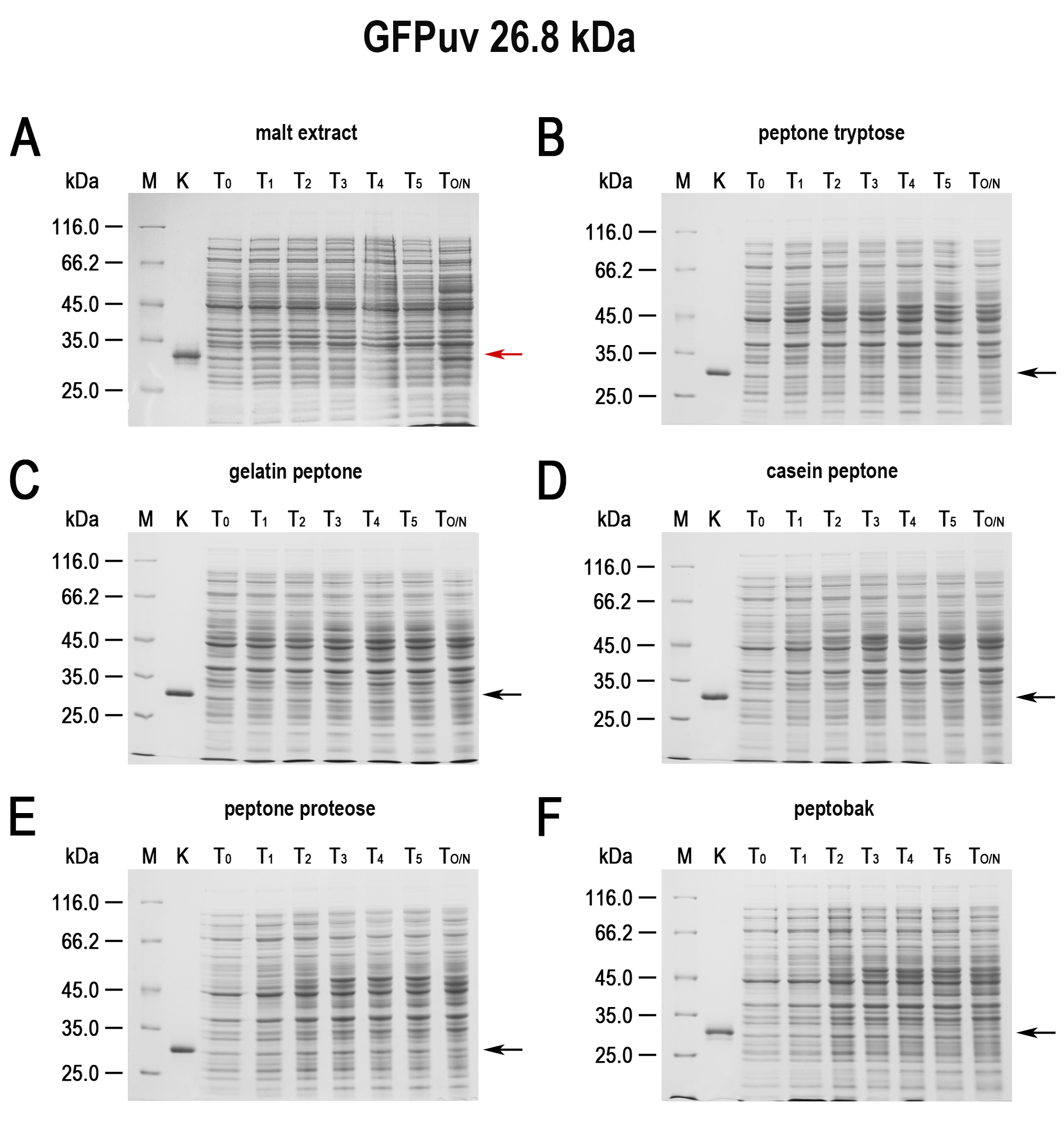

Supplement: Supplementary file 1 — Additional file 1: Expression leakage of GFPuv in E. coli BL21(DE3) [pET21d(+)-gfpuv] cells grown in media containing selected peptones. The cultivation, sample preparation and analysis were identical to that described in Fig. 2, except that a different peptone was supplemented in the composition of the medium instead of the most commonly used soya peptone and tryptone peptone: A wheat extract; B tryptose peptone; C gelatin peptone; D casein peptone; E proteose peptone; and F peptobak. [file 12934_2022_1740_MOESM1_ESM.tif]

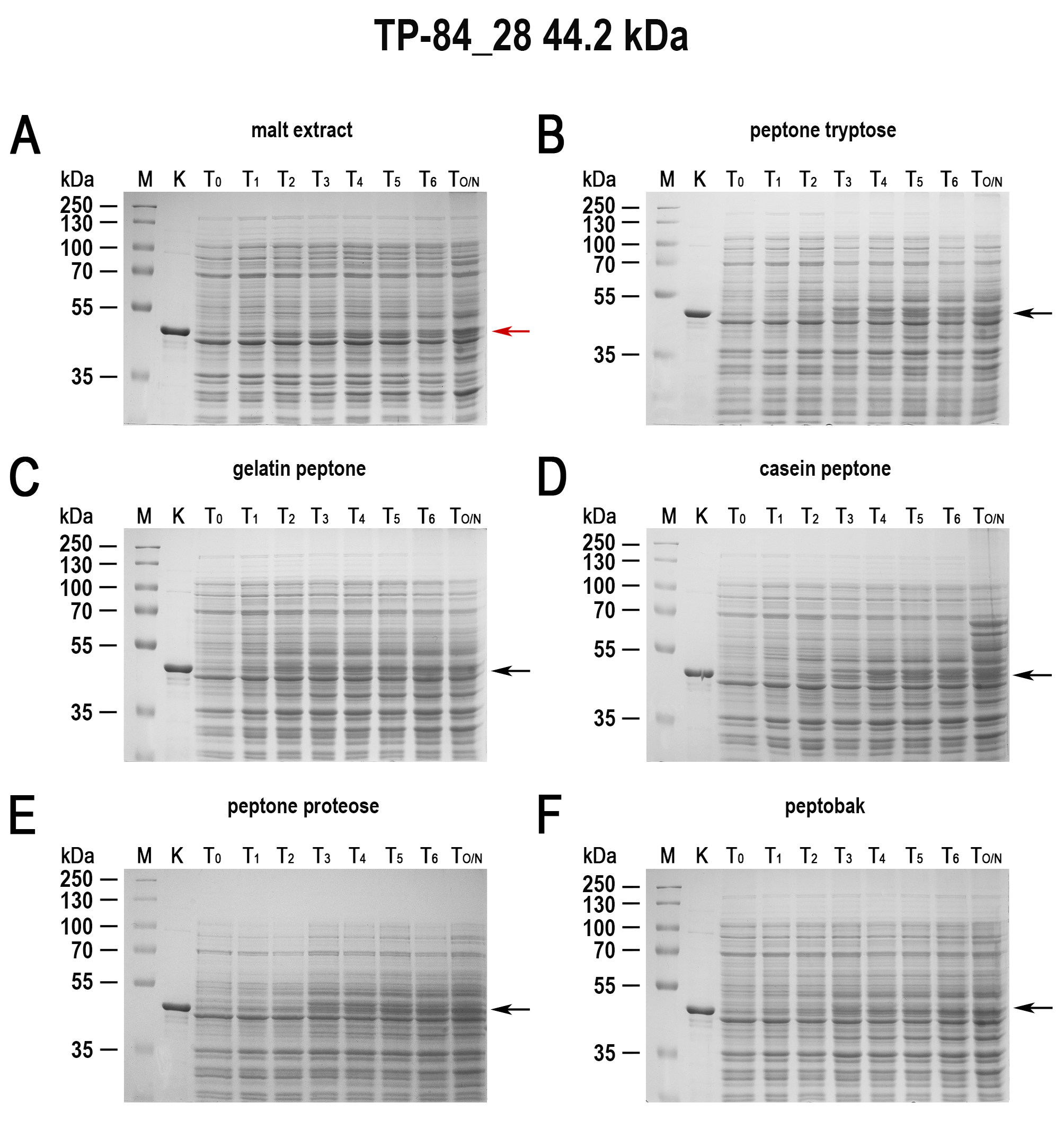

Supplement: Supplementary file 2 — Additional file 2: Expression leakage of TP-84 endolysin in E. coli BL21(DE3) [pET21d(+)-tp-84_28] cells grown in media containing selected peptones. The cultivation, sample preparation and analysis were identical to those described in Fig. 3, except that a different peptone was supplemented in the composition of the medium instead of the most commonly used soya peptone and tryptone peptone: A wheat extract; B tryptose peptone; C gelatin peptone; D casein peptone; E proteose peptone; F peptobak. [file 12934_2022_1740_MOESM2_ESM.tif]

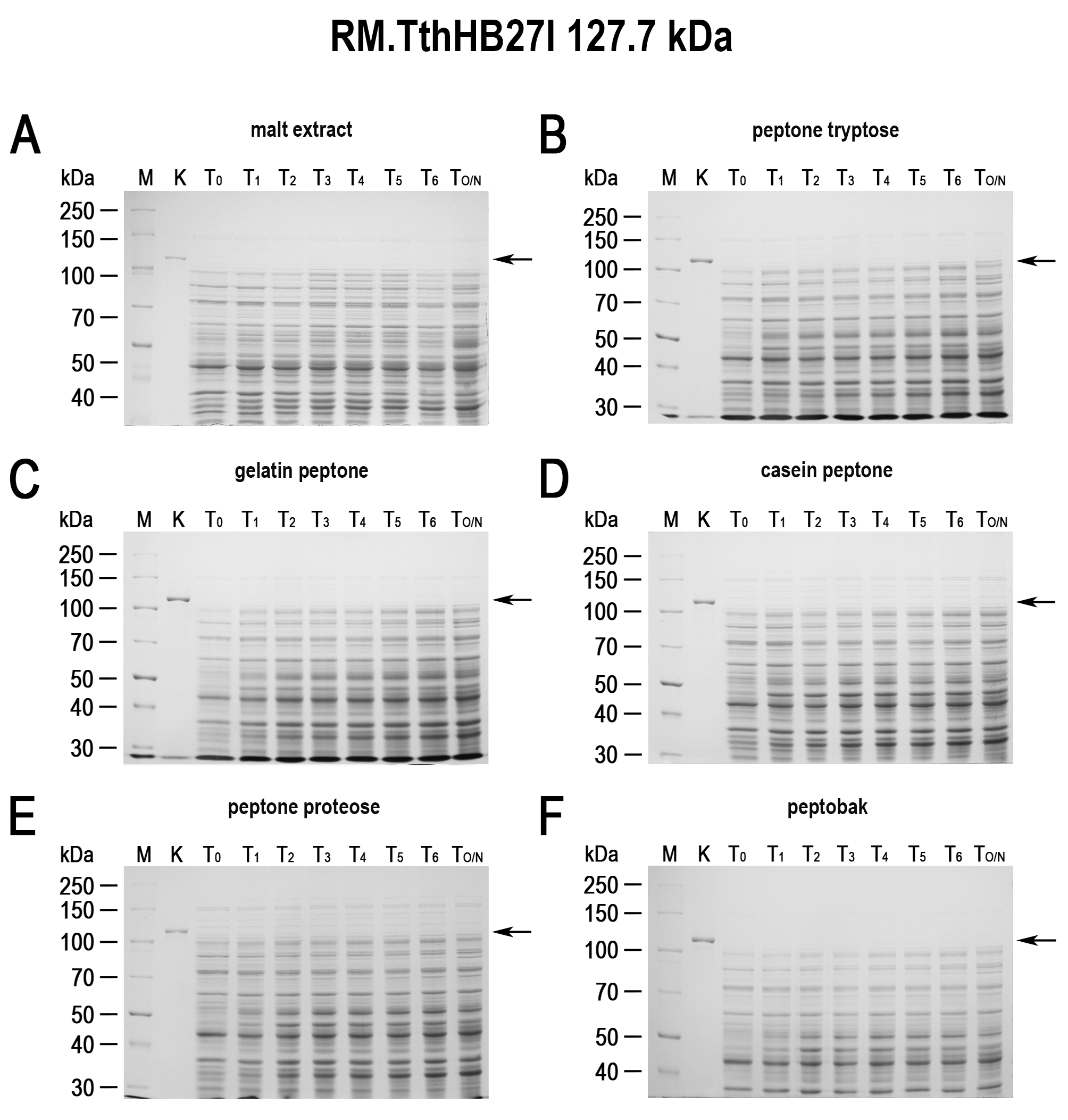

Supplement: Supplementary file 3 — Additional file 3: Expression leakage of RM.TthHB27I in E. coli BL21(DE3) [pET21d(+)-tthHB27IRM] cells grown in media containing selected peptones. The cultivation, sample preparation and analysis were identical to that described in Fig. 4, except that a different peptone was supplemented in the composition of the medium instead of the most commonly used soya peptone and tryptone peptone: A wheat extract; B tryptose peptone; C gelatin peptone; D casein peptone; E proteose peptone; F peptobak. [file 12934_2022_1740_MOESM3_ESM.tif]
