# Supplementary material for: T7-lac promoter vectors spontaneous derepression caused by plant-derived growth media may lead to serious expression problems: a systematic evaluation
Source: Microb Cell Fact. 2022 Jan 28;21:13. doi: 10.1186/s12934-022-01740-5 (PMC8796431; doi:10.1186/s12934-022-01740-5)
Supplement: Supplementary file 4 — Additional file 4: Bacterial culture optical density measurements data. Optical density measurements were performed at a wavelength of 600 nm using sterile medium as a background. [file 12934_2022_1740_MOESM4_ESM.docx]

| **BL21(DE3) [pET21d(+)-*gfpuv*]** | | | | | | | | | | | |
| --- | --- | --- | --- | --- | --- | --- | --- | --- | --- | --- | --- |
| **time [h]** | **T*** | **yeast extract only** | **M9 minimal medium** | **soy peptone** | **malt extract** | **tryptone peptone** | **tryptose peptone** | **gelatin peptone** | **casein peptone** | **proteose peptone** | **peptobak** |
| **0** |  | 0.102 | 0.099 | 0.104 | 0.11 | 0.102 | 0.097 | 0.101 | 0.104 | 0.096 | 0.107 |
| **1** |  | 0.234 | 0.265 | 0.368 | 0.439 | 0.243 | 0.348 | 0.287 | 0.223 | 0.285 | 0.328 |
| **2** | **T_0_** | 0.588 | 0.641 | 0.805 | 1.171 | 0.626 | 0.922 | 0.698 | 0.517 | 0.794 | 0.865 |
| **3** | **T_1_** | 1.316 | 1.182 | 1.934 | 2.624 | 1.492 | 1.758 | 1.398 | 1.37 | 1.676 | 1.908 |
| **4** | **T_2_** | 1.84 | 1.88 | 2.76 | 3.72 | 2.45 | 2.455 | 2.07 | 2.34 | 3.16 | 2.845 |
| **5** | **T_3_** | 1.97 | 2.44 | 3.51 | 5.3 | 3.37 | 3.4 | 3.04 | 3.6 | 3.99 | 4.02 |
| **6** | **T_4_** | 2.03 | 2.69 | 4.45 | 5.46 | 3.41 | 3.81 | 3.58 | 3.79 | 4.52 | 4.26 |
| **7** | **T_5_** | 2.42 | 3.62 | 5.31 | 6.51 | 3.91 | 4.97 | 3.61 | 4.15 | 5.65 | 6.04 |
| **8** | **T_6_** | 2.52 | 3.51 | 6.69 | 6.6 | 4.02 | 5.13 | 3.96 | 4.2 | 6.36 | 6.08 |
| T* - time point at which the culture samples were withdrawn for SDS-PAGE analysis | | | | | | | | | | | |

| **BL21(DE3) [pET21d(+)-*tp84_28*]** | | | | | | | | | | | |
| --- | --- | --- | --- | --- | --- | --- | --- | --- | --- | --- | --- |
| **time [h]** | **M9 minimal medium**  **T*** | **yeast extract only** |  | **soy peptone** | **malt extract** | **tryptone peptone** | **tryptose peptone** | **gelatin peptone** | **casein peptone** | **proteose peptone** | **peptobak** |
| **0** |  | 0.161 | 0.096 | 0.159 | 0.137 | 0.134 | 0.113 | 0.157 | 0.124 | 0.117 | 0.146 |
| **1** |  | 0.423 | 0.33 | 0.431 | 0.304 | 0.313 | 0.289 | 0.398 | 0.258 | 0.283 | 0.297 |
| **2** | **T_0_** | 0.784 | 0.744 | 0.778 | 0.628 | 0.659 | 0.581 | 0.747 | 0.519 | 0.573 | 0.606 |
| **3** | **T_1_** | 1.396 | 1.254 | 1.78 | 1.224 | 1.234 | 1.326 | 1.586 | 1.132 | 1.398 | 1.412 |
| **4** | **T_2_** | 1.305 | 1.924 | 3.08 | 1.626 | 2.17 | 2.448 | 2.145 | 1.596 | 2.61 | 2.538 |
| **5** | **T_3_** | 1.75 | 2.16 | 3.525 | 2.085 | 2.781 | 3.11 | 2.665 | 2.6 | 3.725 | 3.495 |
| **6** | **T_4_** | 1.8 | 2.06 | 4.086 | 2.3 | 2.935 | 3.81 | 3.024 | 2.775 | 4.146 | 4.302 |
| **7** | **T_5_** | 1.92 | 2.65 | 4.722 | 2.645 | 3.18 | 4.506 | 3.246 | 3.35 | 5.184 | 4.68 |
| **8** | **T_6_** | 2.13 | 2.5 | 5.034 | 3.615 | 3.805 | 4.698 | 3.294 | 3.91 | 5.358 | 5.67 |
| T* - time point at which the culture samples were withdrawn for SDS-PAGE analysis | | | | | | | | | | | |

| **BL21(DE3) [pET21d(+)-*tthHB27IRM*]** | | | | | | | | | | | |
| --- | --- | --- | --- | --- | --- | --- | --- | --- | --- | --- | --- |
| **time [h]** | **T*** | **yeast extract only** | **M9 minimal medium** | **soy peptone** | **malt extract** | **tryptone peptone** | **tryptose peptone** | **gelatin peptone** | **casein peptone** | **proteose peptone** | **peptobak** |
| **0** |  | 0.117 | 0.098 | 0.122 | 0.092 | 0.113 | 0.127 | 0.096 | 0.103 | 0.107 | 0.128 |
| **1** |  | 0.243 | 0.279 | 0.252 | 0.205 | 0.21 | 0.276 | 0.2 | 0.234 | 0.213 | 0.282 |
| **2** | **T_0_** | 0.5 | 0.556 | 0.53 | 0.653 | 0.39 | 0.6 | 0.47 | 0.54 | 0.57 | 0.6 |
| **3** | **T_1_** | 0.946 | 1.125 | 1.087 | 1.088 | 0.757 | 1.174 | 0.891 | 1.117 | 1.132 | 1.291 |
| **4** | **T_2_** | 1.71 | 1.528 | 2.59 | 1.659 | 1.65 | 1.77 | 1.68 | 2.15 | 2.28 | 2.33 |
| **5** | **T_3_** | 2.03 | 2.2 | 3.04 | 2.18 | 2.19 | 2.78 | 2.46 | 3.33 | 2.91 | 3 |
| **6** | **T_4_** | 1.97 | 1.87 | 3.49 | 2.595 | 2.68 | 3.23 | 2.8 | 3.45 | 3.21 | 3.6 |
| **7** | **T_5_** | 2.31 | 2.11 | 2.18 | 2.68 | 3.16 | 3.62 | 3.12 | 3.88 | 3.88 | 4.16 |
| **8** | **T_6_** | 2.28 | 2.15 | 0.66 | 3.22 | 3.34 | 3.98 | 3.31 | 4.03 | 4.46 | 4.62 |
| T* - time point at which the culture samples were withdrawn for SDS-PAGE analysis | | | | | | | | | | | |
