# Supplementary material for: T7-lac promoter vectors spontaneous derepression caused by plant-derived growth media may lead to serious expression problems: a systematic evaluation
Source: Microb Cell Fact. 2022 Jan 28;21:13. doi: 10.1186/s12934-022-01740-5 (PMC8796431; doi:10.1186/s12934-022-01740-5)
Supplement: Supplementary file 5 — Additional file 5: Table summarising the basic data on the tested nutrients and price comparison. The price comparison is shown as of the highest-priced component. The prices quoted by the producers of the ingredients were used in this research may vary. [file 12934_2022_1740_MOESM5_ESM.docx]

| **Component** | **Media ingredient brief description** | **BTL** | | **Merck** | |
| --- | --- | --- | --- | --- | --- |
|  |  | **Price [€/kg]** | **Relative price** | **Price [€/kg]** | **Relative price** |
| yeast extract | Dry yeast autolysate. Source of nitrogen and vitamins. | 51.67 | 42.9% | 326.06 | 56.4% |
| malt extract | Malt extract - a rich source of carbohydrates. Used in media for mold and yeast. | 56.94 | 48.6% | 140.5 | 24.3% |
| soy peptone | Papain soybean hydrolysate. A rich source of vitamins and carbohydrates. It can be used for the cultivation of various microorganisms, including the most demanding ones. | 86.58 | 63% | 332.42 | 57.5% |
| peptone tryptone | Pepsin hydrolysate of casein. A mixture of peptides, where the appropriate proportions of nitrogen and the amino acid system provide conditions for the development of various groups of microorganisms. | 95.96 | 70% | 280.99 | 48.6% |
| peptone tryptose | A mixture of peptones with high nutritional properties. A component of microbiological media for the growth of microorganisms with high nutritional requirements. | 137.31 | 100% | 341.43 | 59.1% |
| peptone proteose | Enzymatic hydrolysate of selected animal tissue with a high content of free amino acids. Component of many microbiological media. | 137.31 | 100% | 577.89 | 100% |
| gelatin peptone | Pancreatic gelatin hydrolysate. It is characterized by a low content of sulfur amino acids and a high content of proline and hydroxyproline. | 82.39 | 60% | 271.98 | 47.1% |
| casein peptone | Pancreatic casein hydrolysate. Used in many media for routine microbiological control as well as in media for industrial cultivation of lactic acid bacteria, *Bacillus*, *Brucella*, etc. | 83.24 | 60.6% | 280.99 | 48.6% |
| peptobak | Enzymatic hydrolysate of selected animal tissue containing mainly peptides of average molecular weight. Component of many microbiological media. | 129.10 | 94% | not available | |
